# SINE Insertion in the Pig Carbonic Anhydrase 5B *(CA5B)* Gene Is Associated with Changes in Gene Expression and Phenotypic Variation

**DOI:** 10.3390/ani13121942

**Published:** 2023-06-09

**Authors:** Yao Zheng, Cai Chen, Mengli Wang, Ali Shoaib Moawad, Xiaoyan Wang, Chengyi Song

**Affiliations:** 1 College of Animal Science and Technology, Yangzhou University, Yangzhou 225009, China; mz120180996@yzu.edu.cn (Y.Z.); 007302@yzu.edu.cn (C.C.); mx120200833@yzu.edu.cn (M.W.); ali.shoeib@agr.kfs.edu.eg (A.S.M.); wxyan@yzu.edu.cn (X.W.); 2 International Joint Research Laboratory, Universities of Jiangsu Province of China for Domestic Animal Germplasm Resources and Genetic Improvement, Yangzhou 225009, China; 3 Department of Animal Production, Faculty of Agriculture, Kafrelsheikh University, Kafrelsheikh 33516, Egypt

**Keywords:** pig, RIPs, *CA5B*, enhancer, growth performance

## Abstract

**Simple Summary:**

Carbonic anhydrases (CAs) play a crucial role in numerous physiological and pathological processes in animals. Specifically, carbonic anhydrase VB (CA5B) is integral to both gluconeogenesis and lipogenesis. Structural variations, particularly retrotransposon insertion polymorphisms (RIPs), can contribute significantly to these processes. When an RIP is inserted into a genome region containing functional elements (such as promoters, enhancers, and insulators), it can have an impact on both the expression and phenotypic changes of target genes. In this study, we identified an RIP located in the first intron of the *CA5B* gene, inserted by the SINE element, which enhanced the core promoter activity of the target gene. Through experimental analysis and growth data provided by the pig breeding farm (Anhui academy of agriculture sciences), we found that the SINE insertion improved the expression of the porcine *CA5B* gene in adipose tissue. Additionally, the number of (ATTT) repeats within the SINE insertion at this site was inconsistent across breeds and individuals. This study provides insight into the role RIPs play in genetic variation of *CA5B* genes and its phenotypic expression in pigs.

**Abstract:**

Transposons are genetic elements that are present in mammalian genomes and occupy a large proportion of the pig genome, with retrotransposons being the most abundant. In a previous study, it was found that a SINE retrotransposon was inserted in the 1^st^ intron of the *CA5B* gene in pigs, and the present study aimed to investigate the SINE insertion polymorphism in this gene in different pig breeds. Polymerase chain reaction (PCR) was used to confirm the polymorphism in 11 pig breeds and wild boars), and it was found that there was moderate polymorphism information content in 9 of the breeds. Further investigation in cell experiments revealed that the 330 bp SINE insertion in the RIP-*CA5B* site promoted expression activity in the weak promoter region of this site. Additionally, an enhancer verification vector experiment showed that the 330 bp SINE sequence acted as an enhancer on the core promoter region upstream of the *CA5B* gene region. The expression of *CA5B* in adipose tissue (back fat and leaf fat) in individuals with the (SINE^+/+^) genotype was significantly higher than those with (SINE^+/−^) and (SINE^−/−^) genotypes. The association analysis revealed that the (SINE^+/+^) genotype was significantly associated with a higher back fat thickness than the (SINE^−/−^) genotype. Moreover, it was observed that the insertion of SINE at the RIP-*CA5B* site carried ATTT repeats, and three types of (ATTT) repeats were identified among different individuals/breeds (i.e., (ATTT)_4_, (ATTT)_6_ and (ATTT)_9_). Overall, the study provides insights into the genetic basis of adipose tissue development in pigs and highlights the role of a SINE insertion in the *CA5B* gene in this process.

## 1. Introduction

Carbonic anhydrases (CAs) are a group of metalloenzymes that are found throughout the body and are responsible for various biosynthetic processes such as gluconeogenesis, lipogenesis, and ureagenesis [1]. There are currently 15 subtypes of CA that have been identified in humans, including CA1-14, with CA5 being further divided into the subtypes of *CA5A* and *CA5B* [2,3]. Among these subtypes, three are essential for the entire process of fatty acid biosynthesis: *CA5A* and *CA5B* in mitochondria and CAII in the cytoplasm [4]. *CA5A* is mainly expressed in the liver, skeletal muscle, and kidneys, while *CA5B* is more widely expressed in various other tissues. In particular, *CA5B* is most highly expressed in subcutaneous and genital fat of adult mice [5], where it is involved in the regulation of pyruvate carboxylation and pyruvate gluconeogenesis in adipocytes [6,7].

Retrotransposons, which include short interspersed nuclear elements (SINEs), long interspersed nuclear elements (LINEs), and long terminal repeats (LTRs), are significant components of mammalian transposable elements (TEs) [8,9,10]. They occupy about 37% of the pig genome [11]. Transposon or retrotransposon insertion can cause various genetic effects, such as the activation or inactivation of host genes [12,13]. The mechanism behind these genetic effects may involve the introduction of new functional regulatory elements or the influence of transcriptional and epigenetic regulation [14]. Both DNA transposon and retrotransposon insertions have been observed to cause phenotypic variations, as seen in the 8.2 kb PiggyBac-derived insertion, which leads to a trans-species color polymorphism in Midas cichlid fishes [15], and the 7.5 kb retrotransposon insertion, including the complete endogenous retroviruses (ERVs), into the 5’UTR of the CYP19A1 gene in hens that result in feather phenotypic variation [16].

SINE, a short fragment of retrotransposon, requires the mediation of the LINE retrotransposon to achieve transposition. For SINE RNA to be successfully inserted into the target genome, a series of processes such as splicing, annealing, and reverse transcription is required, resulting in 2–15 bp target site duplication (TSD) on both ends at the insertion site [17]. Compared to LINE and LTR, SINE has the most widespread distribution in mammalian genomes [18] and may have the most significant genomic and genetic impacts. Extensive reports suggest that SINE retrotransposon insertions can cause genetic and phenotypic variations in mammals. For example, phenotypic variations derived from SINE insertion have been observed in dogs for both body size [19] and coat coloration [20]. In pigs, SINE insertions are also associated with variations in economic traits and gene expression [21,22]. Although the knowledge about porcine repetitive element 1 (PRE-1)—the pig SINEA1 element—is limited compared to that in primates, specific features of PRE-1 make pigs a compelling model for further investigation. PRE-1 currently has the third highest copy number of any catalogued SINE in SINE-Base [11,23,24,25].

In our previous study, we found that over 65% of putative SINE insertion polymorphisms (RIPs) overlap with genes, and 51.36% of these overlap with protein-coding genes. Furthermore, 4954 SINE RIPs were observed to be located in the first introns of protein-coding genes, with one SINE RIP reported in the first intron of the *CA5B* gene [26]. Therefore, the present study aimed to investigate a SINE insertion polymorphism in the first intron of the *CA5B* gene across various pig breeds, using polymerase chain reaction (PCR), and to determine whether there is any association between the phenotype and the SINE insertion in the breeds included in our study.

## 2. Materials and Methods

### 2.1. Animals and Sampling

A total of 754 animals from 11 domesticated pig breeds and wild pigs were used for genotyping. Table 1 summarizes the type, origin, sample size, and breeds that were crossed to create these breeds.

### 2.2. DNA Isolation and Amplification 

Ear tissue samples were used to extract DNA using the TIANamp Genomics DNA Kit (TIANGEN, Beijing, China) according to the manufacturer’s instructions. The genomic flank regions of the SINE insertion site served as a basis to design PCR primers using Oligo7.0 software, as listed in Supplementary Table S1. The PCR amplification reaction (20 μL) consisted of 50 ng genomic DNA, 10 pmol of upstream and downstream primers, and 10 μL 2× TaqMix (Vazyme, Nanjing, China). Enzyme-free water was added to make up the 20 μL. The PCR reaction included pre-denaturation at 95 °C for 5 min, followed by 30 cycles of denaturation at 95 °C for 30 s, annealing at the primer’s corresponding annealing temperature for 30–60 s, and extension at 72 °C for 30 s. Finally, there was a further extension at 72 °C for 5 min and storage at 4 °C. Electrophoresis was used to assess the PCR product on a 1.5% agarose gel soaked in an ethidium bromide staining solution for 20 min. The gel was then put in an automatic digital gel image analysis system for photography.

### 2.3. RIP Verification and Genotyping by PCR

DNA samples from 11 domestic pig breeds and wild boars were used to verify the RIP in the *CA5B* gene via PCR. Two DNA pools per breed and for wild boars were used for PCR amplification. Each pool contained three different individuals with equal DNA concentrations. Population analysis of the RIP in *CA5B* was conducted using nine breeds, namely Duroc, Landrace, Large White, Sushan, Fengjing, Meishan, Erhualian, Sujiang, and Bama. For this analysis, 24 individuals were genotyped from each breed, with the exception of Sushan, which had 32 individuals.

### 2.4. Economic Trait Data Collection 

Growth performance indicators, including body weight, back fat thickness, and eye muscle thickness, were collected as vital growth performance predictors for 482 Large White pigs. The body weight of the pigs was measured using an electronic scale, and the age at which the weight reached 100 kg was recorded for correction. Back fat thickness was measured between the 3rd and 4th ribs, 5.0 cm on the side of the backline when the pig’s weight reached 100 kg, using the LX 8000 B ultrasonic device (Beijing Kangchengda Technology Co., Ltd., Beijing, China).

### 2.5. Expression Analysis

To quantify the expression levels of *CA5B*, Sushan pigs aged 180–200 days were genotyped, and three pigs with each genotype (SINE^+/+^, SINE^+/−^, and SINE^−/−^) were selected and slaughtered to collect tissue samples, including liver, longissimus dorsi, leg muscle, back fat, and leaf fat. Total RNA was extracted from these tissues using the Trizol kit (TIANGEN, Beijing, China), and cDNA was synthesized using the FastKing kit (TIANGEN, Beijing, China) according to the manufacturer’s instructions. RT-qPCR was performed using the ChamQ Universal SYBR qPCR Master Mix (Vazyme, Nanjing, China) kit and a fluorescence quantitative detector of qTower3 G (Analytik Jena AG, Thuringia, Germany) following the recommended protocol. The reaction mixture contained 1 μL cDNA template, 10 μL 2× ChamQ Universal SYBR qPCR Master Mix (Vazyme, Nanjing, China), 0.4 μL upstream and downstream primers (10 μmol/μL), and 8.2 μL deionized water. Each sample was run in triplicate, and precautions were taken to avoid exposure to light and maintain a low temperature throughout the experiment. The PCR conditions comprised an initial denaturation at 95 °C for 30 s, followed by 40 cycles of denaturation at 95 °C for 3 s, extension at 60 °C for 30 s, and a final cooling at 12 °C. Pig β-actin was used as an endogenous positive control. The relative expression of the gene was calculated using the 2^−△△CT^ formula, and the results were expressed as mean ± standard error. The accuracy of the RT-qPCR experiment was confirmed by analyzing the dissolution curve of the detection results. All the RT-qPCR primers used are listed in Supplementary Table S1.

### 2.6. Vector Construction

To assess the potential promoter and enhancer activity of the SINE insertion in the *CA5B* gene, two fragments of the insertion were cloned. The first fragment, referred to as *CA5B*^SINE+389bp^, consisted of the SINE insertion (330 bp) along with a 59 bp genomic flank (NC_010461.5:12298865-12298923), resulting in a total length of 389 bp. The second fragment, referred to as *CA5B*^SINE+783bp^, included 453 bp genomic flanks (NC_010461.5:12298630-12299082), resulting in a total length of 783 bp. These fragments were each inserted into the PGL3-Enhancer vector (Promega, Madison, WI, USA) to construct *CA5B*^SINE+389bp^-LucEn and *CA5B*^SINE+783bp^-LucEn vectors, respectively. Additionally, two control vectors were constructed by cloning and inserting the wild type genomic flanks of either 59 bp (named as *CA5B*^SINE-59bp^) or 453 bp (named as *CA5B*^SINE-453bp^) into the same PGL3-Enhancer vector.

To further confirm the enhancer activity of the SINE insertion, mini-promoters for Oct4 and Myc genes were cloned from the pTol2-Oct4-mCherry and pTol2-Myc-mCherry vector [27], respectively. These mini-promoters were then inserted into the PGL3-basic vector to create two new vectors—Oct4-Luc and Myc-Luc. Next, the SINE insertion (330 bp) without the adjacent flank sequence, referred to as SINE+, was inserted into both the Oct4-Luc and Myc-Luc vectors. The resulting vectors were named *CA5B*^SINE+^-Oct-Luc and *CA5B*^SINE+^-Myc-Luc, respectively.

In addition, we cloned and inserted two putative core promoter regions, *CA5B*-pro1 (NC_010461.5:12293450-12294070, 621 bp) and *CA5B*-pro2 (NC_010461.5:12295050-12295585, 505 bp), into the PGL3-basic vector to construct the *CA5B*-pro1-Luc and *CA5B*-pro2-Luc vectors, respectively. These core promoter regions were predicted using BDGP https://www.fruitfly.org/seq_tools/promoter.html (accessed on 30 November 2022) and Promoter 2.0 https://services.healthtech.dtu.dk/service.php?Promoter-2.0 (accessed on 30 November 2022). To create two SINE-enhanced vectors, *CA5B*-pro1^SINE+^-Luc and *CA5B*-pro2^SINE+^-Luc, the SINE insertion (330 bp) without the adjacent flank sequence (SINE+) was inserted into the upstream regions of *CA5B*-pro1 and *CA5B*-pro2, respectively.

During the experiment, we used several control vectors, including PGL3-control (containing the SV40 promoter and enhancer from Promega, Madison, WI, USA), PGL3-Enhancer (containing the SV40 enhancer from Promega, Madison, WI, USA), PGL3-basic (from Promega, Madison, WI, USA), β-globin-Luc, and Oct4-Luc. Supplementary Table S1 provides a list of all the primers used during the cloning process.

### 2.7. Dual-Luciferase Reporter Assay

For cultivating 3T3-L1 and PK-15 cells, a total of 2 × 10^5^ cells were transferred to 6-well plates and transfected using transfection reagents to construct clones. Plasmids were transfected separately into the cultured 3T3-L1 and PK-15 cells, and after 48 h, the luciferase activity was evaluated using the dual-luciferase reporter system (Vazyme, Nanjing, China) as per the manufacturer’s instructions with a Modulus™ II Microplate Multimode Reader (Turner Biosystems, Sunnyvale, CA, USA). For each experiment, we repeated the process thrice and performed three independent experiments. PK15 and 3T3-L1 cells were cultured in DMEM medium containing 10% fetal bovine serum, 100 U/mL penicillin, and 0.1 mg/mL streptomycin. We maintained all the cells in a humidified atmosphere with 5% CO_2_ in the air at 37 °C, and we purchased all the cell culture reagents from Thermo Fisher Scientific (Waltham, MA, USA). We processed the experimental results statistically using the SPSS17.0 software package (SPSS, Inc., Chicago, IL, USA) with the T-test, and we expressed the data as mean ± standard error.

### 2.8. Cloning and Sequencing of the SINE-Carried (ATTT)_n_ Repeat Sequence across Genomes

We cloned and sequenced the SINE insertion of RIP-*CA5B* including flanks from different genomes. The primer for this experiment was synthesized and sequenced by the Tsingke company (Tsingke, Nanjing), and we listed it in the Supplementary Table S1. We also genotyped and selected at least 5 individuals with RIP-*CA5B* locus of SINE insertion type from 9 different breeds of pigs. The 9 pig breeds included Duroc, Large White, Landrace, Sujiang, Bama, Erhualian, Meishan, Sushan, and Tibetan. We then cloned and sequenced the selected individuals at the RIP-*CA5B* locus, and we aligned the sequence results using the ClustalW Multiple alignment programs in the Bioedit software to determine the repeat number of the ATTT sequence carried by the SINE at the RIP-*CA5B* locus.

### 2.9. Statistical Analysis and Growth Correlation Analysis

We used SPSS 17.0 for statistical analysis of the growth performance indicators data. The data results were expressed as mean ± standard error, and we used Duncan’s multiple range test to compare the differences between groups. For population analysis, we used POP-GENE to detect the Hardy–Weinberg equilibrium, and we calculated the polymorphic information content (PIC) using the following formula:

$$\mathrm{PIC}=1-\overset{m}{\underset{i=1}{\sum }}p_{i}^{2}-\overset{m-1}{\underset{i=1}{\sum }}\overset{m}{\underset{j=i+1}{\sum }}2P_{i}^{2}P_{j}^{2}$$

where, *P_i_* and *P_j_* are the frequencies of the *i*^th^ gene and the *j*^th^ allele in the population, respectively; *m* is the number of alleles.

## 3. Results

### 3.1. SINE RIP in the First Intron of Pig CA5B Gene Verified by PCR and Sequencing 

**Figure 1:**
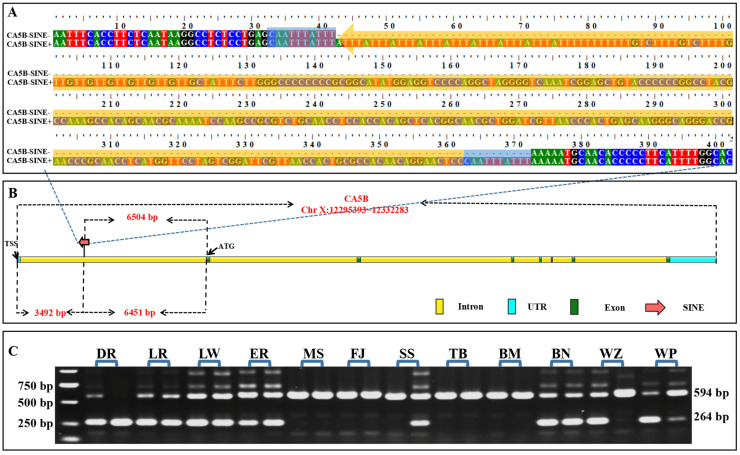
SINE RIP in the first intron of pig *CA5B* gene verified by PCR and sequencing. (**A**) The sequences in RIP-*CA5B* site with and without SINE insertion, the sequence covered by the yellow shadow is 330 bp SINE insertion, while the sequence covered by the blue shadow is the TSD sequence of SINE. (**B**) RIP-*CA5B* location on *CA5B* gene. (**C**) Genotyping of RIP-*CA5B* locus in DNA pools. DR—Duroc; LR—Landrace; LW—Large White; ER—Erhualian; MS—Meishan; FJ—Fengjing; SS—Sushan; TB—Tibetan; BM—Bama; BN—Banna; WZ—Wuzhishan; WP—wild boar.

In a previous study, a structural variant was predicted in the first intron of *CA5B*. The variant was inserted at position 12298877-12298878 relative to the reference genome chromosome X (Sscrofa 11.1: NC_010461.5), and it was generated by SINE insertion according to bioinformatic analysis [26]. To validate this SINE RIP, we used genomic DNA samples from 11 different domestic and pig breeds wild boars to perform PCR and TA cloning sequencing in the current study. The PCR amplification and sequencing results revealed that the structural variant was generated by a 330 bp SINE insertion in the reverse orientation (Figure 1A). The SINE insertion is located at 3492 bp downstream from the transcription start site (TSS) and 6451 bp and 6504 bp upstream from exon 2 and translation start site (ATG), respectively (Figure 1B). Across the breed DNA samples, we observed three genotypes, namely SINE^+/+^, SINE^+/−^, and SINE^−/−^, as shown in Figure 1C.

### 3.2. RIP Distribution in Different Pig Breeds

We used a total of nine pig breeds to characterize the polymorphic distribution in different populations. The breeds included four lean-type breeds (Duroc, Landrace, Large White, and Sushan), three fat-type breeds (Fengjing, Meishan, and Erhualian), one medium-type breed (Sujiang), and one miniature (Bama). In all the breeds except Landrace and Meishan, we detected three genotypes, namely SINE^+/+^, SINE^+/−^, and SINE^−/−^, and we observed differential distribution in these populations (Supplementary Figure S1). In general, we observed high frequencies of SINE^+/+^ genotype and SINE^+^ allele in all the breeds, except Sushan, which displayed high frequencies of SINE^−/−^ genotype and SINE^−^ allele. We found that Hardy–Weinberg equilibrium occurred in only five populations, which were Landrace, Sujiang, Fengjing, Meishan, and Erhualian. We observed that all detected breeds had moderate polymorphism, with PIC values ranging from 0.218 to 0.373, except for Bama, which had a low PIC value of 0.141 (Table 2).

### 3.3. Increased CA5B Expression in Fat Tissues Associated with the SINE-RIP in the First Intron of the CA5B Gene

**Figure 2:**
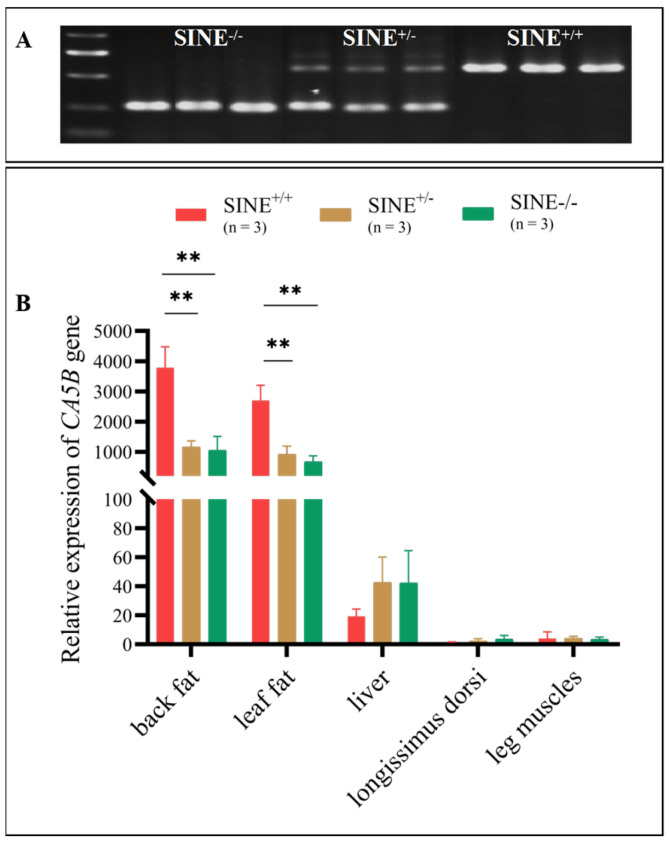
Association of SINE insertion genotype with the expression of *CA5B* in the tissue of adult pigs. (**A**) Genotype result for 9 Sushan pig individuals. (**B**) The results of the relative expression of the *CA5B* gene in different tissues. ** *p* < 0.01.

To further investigate the regulatory role of SINE insertion in the pig *CA5B* gene, we utilized qPCR to analyze differences in gene expression among different genotypes (SINE^+/+^, SINE^+/−^, and SINE^−/−^) of Sushan pigs. Initially, we genotyped 50 Sushan pigs (aged 180–200 days) via PCR, and then selected three individuals for each genotype for slaughter and tissue collection (Figure 2A). Our qPCR findings confirmed a significant association between SINE insertion and *CA5B* gene expression in back fat and leaf fat tissues. Specifically, the expression of the *CA5B* gene in the back fat and leaf fat tissues of SINE^+/+^ pigs was significantly higher than that of SINE^+/−^ and SINE^−/−^ pigs (*p* < 0.05). These results were not evident in the liver, where expression was not statistically different across genotypes, nor in the longissimus dorsi and leg muscles where expression levels were exceedingly low (Figure 2B).

### 3.4. Impact of SINE Insertion in the First Intron of CA5B Gene on Regulatory Activity 

**Figure 3:**
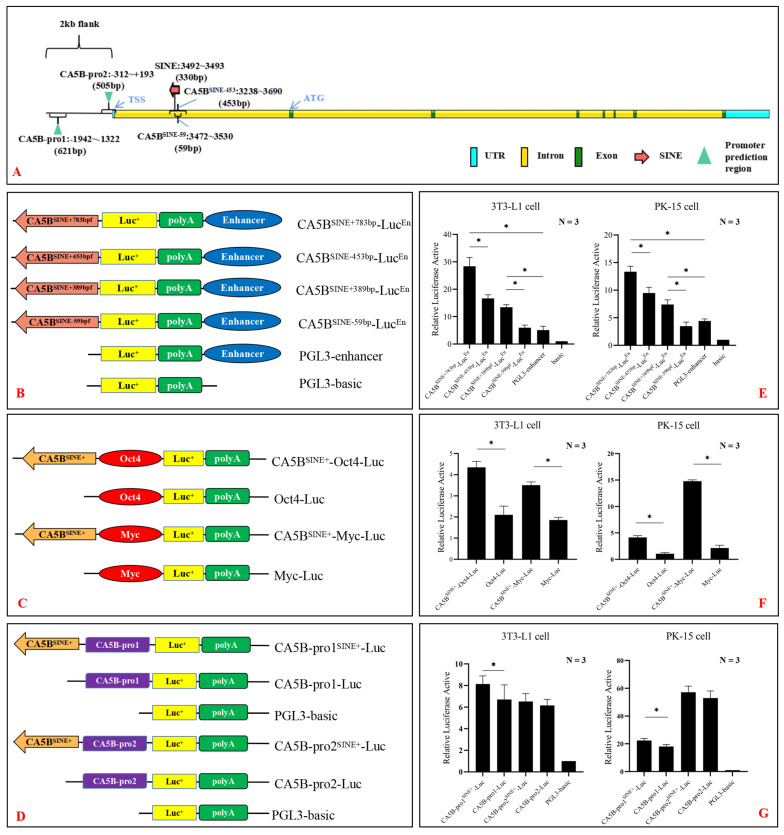
Effect of the 330 bp SINE insertion on the regulation of *CA5B* promoter activity. (**A**) The position of the promoter region in the *CA5B* gene was predicted, and the insertion position of 330 bp SINE was between the 3492-3493 bases. (**B**) Schematic diagram of the vector constructed based on PGL3-enhancer vector to explore the effect of the 330 bp SINE insertion on promoter activity. (**C**) Schematic diagram of the vector constructed based on enhancer verification vectors (Oct4-Luc and Myc-Luc) to explore the effect of the 330 bp SINE insertion on the activity of the weak promoter. (**D**) The vector diagram of the effect of 330 bp SINE insertion on the activity regulation of *CA5B* gene core promoter. (**E**–**G**) The results of the corresponding vectors in B, C and D by dual-luciferase reporter assay, and the number of samples in each experimental group was ran the analyses in triplicat (N = 3); * *p* < 0.05.

To evaluate the impact of a SINE insertion in the first intron of the *CA5B* gene on its regulatory activity, a dual-luciferase reporter assay was used. Two genomic DNA fragments containing the SINE insertion were cloned and sequenced: a long (783 bp) and a short (389 bp) fragment, which were inserted into the PGL3-Enhancer vector for promoter activity evaluation. Two wild-type genomic DNA fragments (453 bp and 59 bp, with no SINE insertion) were used as controls (Figure 3A). The resulting vectors were named *CA5B*^SINE+783bp^-LucEn, *CA5B*^SINE+389bp^-LucEn, *CA5B*^SINE-453bp^-LucEn, and *CA5B*^SINE-59bp^-LucEn, respectively (Figure 3B). The dual-luciferase reporter assay showed that the expression of *CA5B*^SINE+389bp^-LucEn was significantly higher than that of the negative control (PGL3-Enhancer vector) and *CA5B*^SINE-59bp^-LucEn in both 3T3-L1 and PK-15 cells. Similarly, the expression of *CA5B*^SINE+783bp^-LucEn was significantly higher than that of the negative control and *CA5B*^SINE-453bp^-LucEn in both cell lines (Figure 3E). The expression of *CA5B*^SINE-453bp^-LucEn was significantly higher than that of the negative control, while no expression difference was observed between *CA5B*^SINE-59bp^-LucEn and the negative control (Figure 3E). These results suggest that the SINE insertion may exert both promoter and enhancer activities. 

To investigate the putative enhancer activity of the SINE insertion, two mini-promoters (*Oct4* and *Myc*) were cloned and inserted into the PGL3-basic vector for enhancer activity testing [27]. The 330 bp SINE insertion fragment was then cloned and inserted upstream of the mini-promoters in vectors named *CA5B*^SINE+330bp^-Oct4-Luc and *CA5B*^SINE+330bp^-Myc-Luc, respectively (Figure 3C). The dual-luciferase reporter assay showed that the expressions of *CA5B*^SINE+330bp^-Oct4-Luc and *CA5B*^SINE+330bp^-Myc-Luc were significantly higher than those of the negative controls (Oct4-Luc and Myc-Luc) in both 3T3-L1 and PK-15 cells (Figure 3F). These results suggest that the SINE insertion may have enhancer activity for both *Oct4* and *Myc* promoters.

To further explore the enhancer activity of the SINE insertion, the predicted core promoters of the *CA5B* gene were used as targets. Two strong promoter signals in the upstream, named *CA5B*-pro1 (NC_010461.5:12293450-12294070, BDGP: score > 0.9, Promoter 2.0: score > 1.2) and *CA5B*-pro2 (NC_010461.5:12295050-12295585, BDGP: score > 0.8, Promoter 2.0: score > 1.2), were predicted (Figure 3A), and then cloned and inserted into the PGL3-basic vector. The SINE was inserted upstream of both promoters, and the resulting vectors were named *CA5B*-pro1^SINE+^-Luc and *CA5B*-pro2^SINE+^-Luc (Figure 3D). The dual-luciferase reporter assay showed that the expression of *CA5B*-pro1^SINE+^-Luc was significantly higher (*p* < 0.05) than that of *CA5B*-pro2^SINE+^-Luc in both cell lines. However, the expression between *CA5B*-pro2^SINE+^-Luc and *CA5B*-pro2-Luc was not significantly different (Figure 3G). These results demonstrate that the SINE insertion may have enhancer activity for the predicted core promoter *CA5B*-pro1 but not for *CA5B*-pro2.

### 3.5. Growth Association Analysis

The association of the SINE insertion in the first intron of the *CA5B* gene with growth traits was investigated in 482 Large White pigs. Three genotypes (SINE^+/+^, SINE^+/−^, and SINE^−/−^) were detected, with high frequencies of the SINE^+/+^ genotype and SINE^+^ allele observed, in agreement with population analysis of lean-type pig breeds (Table 3). A one-way ANOVA analysis showed that the SINE RIP in the first intron of the *CA5B* gene was significantly associated with back fat thickness (*p* < 0.05). Pigs with the SINE^+/+^ genotype had a significantly higher back fat thickness at 100 kg body weight than those with the SINE^−/−^ genotype. These results suggest a potential role for the SINE insertion in the regulation of fat deposition in pigs.

### 3.6. Length Variations of SINE-carried ATTT Repeat Sequence across Genomes

**Figure 4:**
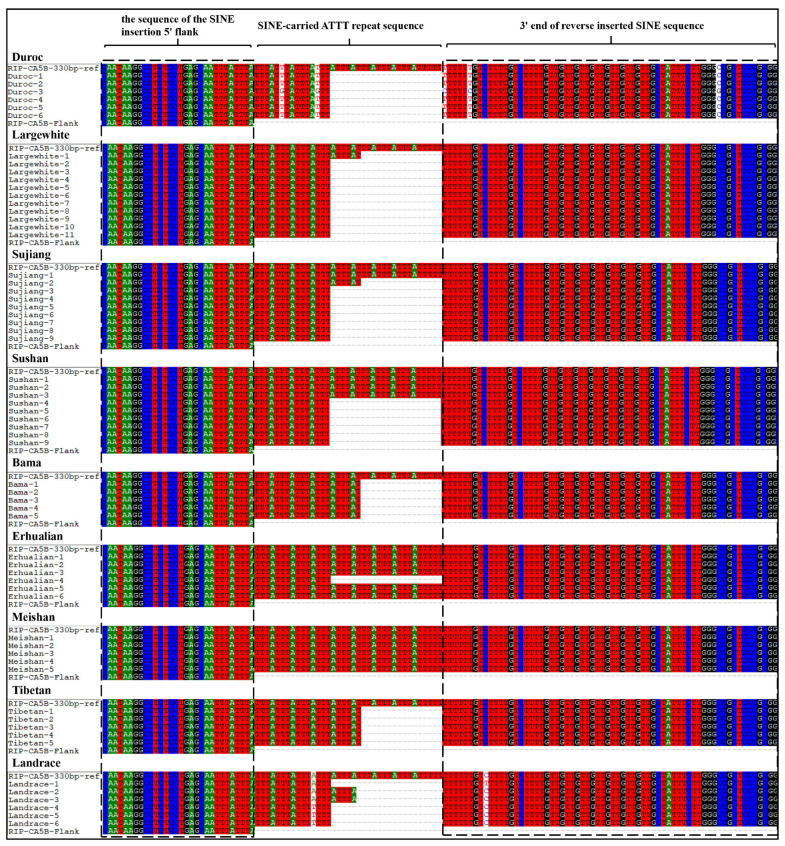
The sequence variation carried by the SINE RIP in *CA5B*. RIP-*CA5B*-330 bp-ref is the sequence of 330 bp SINE inserted into RIP-*CA5B* locus and 5’ flanking 30 bp.RIP-*CA5B*-Flank is the sequence of the RIP-*CA5B* locus and 5’ flanking 30 bp without SINE inserted.

A total of 62 pigs from nine breeds were screened for the SINE insertion in the RIP-*CA5B* locus. The sequencing results of the RIP-*CA5B* locus are shown in Figure 4, and the SINE insertion length variation in individuals of different breeds is shown in Table 4. Alignment of the sequencing results to the sequence of RIP-*CA5B*-330 bp-ref (RIP-*CA5B* locus with 330 bp SINE insertion) revealed different lengths of SINE insertions in different pig breeds and individuals, with different numbers of ATTT repeat sequences found at the 3′ end of the inserted SINE. This resulted in four different sizes of sequence variation (SV) at the RIP-*CA5B* locus. Table 5 shows that SV lengths were associated with differences in the number of (ATTT)_n_ repeats, with three different types identified: (ATTT)_4_, (ATTT)_6_, and (ATTT)_9_. The SINE retrotransposon-derived SV with 308 bp length was repeated four times, representing the (ATTT)_4_ type. SV lengths of 314 bp and 313 bp were of the (ATTT)_6_ type. SV length of 330 bp represented the (ATTT)_9_ type. The majority of the sequenced pigs (55%) carried (ATTT)_4_, followed by (ATTT)_6_ (22%) and (ATTT)_9_ (23%) types. These results indicate that the length and variation of the SINE insertion in the RIP-*CA5B* locus differs among pig breeds and individuals, and is associated with differences in (ATTT)n repeat numbers, revealing potential genetic diversity in this locus across pig populations.

## 4. Discussion

The RIP-*CA5B* locus, under examination in this study, originated from a previously acquired SINE-RIP in our earlier research [26]. In our current research, we have confirmed RIP-*CA5B* as a structural variant site due to the insertion of SINEA1. The SINEA1 is the newest subfamily type in the group of SINE retrotransposons. Previous studies by Chen et al. [26] have demonstrated that SINEA1 has a greater tendency towards RIPs within and between genes in the pig genome. This confirms that the newer SINEs have the potential to produce more RIPs. Similar evidence was found in the dog genome, where the newest SINEC_Cf transposon was more prone to polymorphism [28], which is consistent with our findings.

In our study, we observed a polymorphic insertion of a SINE in the 1st intron of the porcine *CA5B* gene. The presence of this insertion was detected through bioinformatics and was confirmed through PCR experiments, indicating that this site is indeed polymorphic. Furthermore, a population genetic analysis based on genotyping of RIP-*CA5B* in different pig populations showed that polymorphisms were present in all populations with moderate polymorphic information content. Moreover, more than 50% of the population was in Hardy–Weinberg equilibrium. These results confirm that the RIP-*CA5B* locus is a useful marker for genetic analysis of pigs. However, four out of the nine tested breeds did not conform to the Hardy–Weinberg equilibrium (Duroc, Large White, Sushan, and Bama). We have justifiable explanations for this issue. For instance, Duroc, Large White, and Sushan are commercial breeding breeds that undergo directional selection, which can lead to nonconformity with the Hardy–Weinberg equilibrium. Additionally, Bama is a relatively small, local Chinese pig breed with a limited population size and non-random mating patterns, which also causes departures from the Hardy–Weinberg equilibrium.

When a retrotransposition event occurs in the genome, retrotransposons can influence gene regulation by introducing their sequences into the regulatory regions of genes, including promoters, cryptic splice sites, terminators, enhancers, and insulators [29]. The regulatory elements introduced by retrotransposons can interfere with gene expression and structure in genes located near or overlapping with the retrotransposon insertion site [30]. Based on Figure 3E, the conducted experiment demonstrated that the 453 bp sequence displayed low promoter activity in the absence of a 330 bp SINE insertion within the RIP-*CA5B* locus. Typically, the weak promoter region is situated prior to the gene’s transcription start site [31]. However, the RIP-*CA5B* site is located within the first intron of the *CA5B* gene, despite the gene having two transcripts annotated in the NCBI database. The location of RIP-*CA5B* is not an ideal region for a strong promoter, although it cannot be solely determined based on the two annotated transcripts of this gene. The NCBI database has identified the position of ATG of this gene downstream from the RIP-*CA5B* site, separated by a distance of 6504 bp. The gene may have a third or even more transcripts at specific phases or in certain tissues. As a result, the RIP-*CA5B* site is likely located upstream from the 5’UTR of the unidentified or undiscovered transcript and acts as a promoter for gene transcription [18].

Our experimental findings, as illustrated in Figure 3E, demonstrate that the 330 bp SINE insertion can enhance host gene regulation and expression, acting as an enhancer element. Additionally, we employed the verification vector (Oct4 Luc and Myc Luc) with the enhancer effect expression frame, as shown in Figure 3C, to confirm that the 330 bp SINE has a specific enhancer effect at this site. Based on our analysis, shown in Figure 3G, we predict that both *CA5B* core promoters have a promoting effect, and the 330 bp SINE can increase the expression of *CA5B*-pro1 (621 bp), but it does not contribute to the expression of *CA5B*-pro2 (505 bp). In the pig genome, the SINE insertion of RIP-*CA5B* may enhance the promoter effect of the host gene -1942~1322 region in some way, and facilitate transcriptional regulation of the *CA5B* gene. Similarly, Chen et al. [21] reported that a SINE retrotransposon insertion in the first intron of the porcine GHR gene generated a polymorphism. Cell experiments also confirmed that the SINE retrotransposon insertion could affect the GHR gene promoter activity. Based on the results of the cell experiments, we have hypothesized that the SINE insertion sequence has the potential to act as both a promoter and an enhancer, and can affect gene expression and phenotype to some extent.

In the present investigation, real-time fluorescent PCR detection technology was utilized to assess the expression of three genotypes produced by RIP-*CA5B* in Sushan pigs. The study found that the expression of *CA5B* in adipose tissue “back fat and leaf fat” significantly increased in individuals with the (SINE^+/+^) genotype compared to those with (SINE^+/−^) and (SINE^−/−^) genotypes. These findings suggest that the insertion of the SINE transposon increased the activity of *CA5B* gene transcription, leading to an increase in the production of carbonic anhydrase-related proteins. These proteins play a crucial role in numerous biosynthetic processes in the body, such as gluconeogenesis and lipogenesis. This can be attributed to the higher transcriptional activity of the gene following the insertion of the SINE transposon into the pig *CA5B* gene. The growth performance and polymorphism of RIP-*CA5B* were investigated in 482 Large White pigs, which revealed that the RIP-*CA5B* genotype was significantly associated with back fat thickness (*p* < 0.05), with the SINE^+/+^ genotype having a significantly higher back fat thickness at 100 kg body weight than the SINE^−/−^ genotype. The effect of SINE insertion on *CA5B* expression and phenotype may have other specificities. Therefore, additional studies are needed to explore the genotypic and phenotypic effects of RIP-*CA5B* on pigs.

According to the results of cloning and sequencing, the SINE insertion of the RIP-*CA5B* contained ATTT repeat sequences that led to variations in base numbers both within and among pig breeds, resulting in different SV lengths across individuals. As per Table 4 and Table 5 and Figure 4, SINE insertion at the RIP-*CA5B* locus carried three cases of ATTT repeat sequences. These differences were not only observed between the breeds but also among individuals within the breeds. The ATTT repeat sequence type (ATTT)_4_ was the most common (56%) among the 64 pigs that were sequenced, exceeding the total of the other two types. The results indicated that the ATTT sequence repeat 4 naturally occurs in the SINE insertion at the RIP-*CA5B* site. The limited number of sequenced individuals may explain why no (ATTT)_4_ individuals were detected in Bama, Meishan, and Tibetan breeds. Likewise, Xu et al. [32] found SINE insertions carrying ATTT repeats in the silkworm genome, and identified SINEs carrying copies of ATTT repeats distributed in its genome. In the present study, the ATTT sequence represents a SINE-derived repeat and is directly associated with SINE. Since the SINE at the RIP-*CA5B* locus is in reverse orientation to the insertion of the *CA5B* gene, the Poly(A) structure at the 3′ end of the SINE is directly attached to the antisense repeat sequence (AAAT)_n_ of (ATTT)_n_. Kosushkin analyzed dog SINE elements and found that young Can_b2 elements tend to carry tandem repeats of TAAA, TAAAA, or TAAAAA at their 3′ end, which affect the target site duplication (TSD) sequences, causing them to start with TAAA, TAAAA, or TAAAAA. This is consistent with our analysis (TSD sequence: CAATTTATTT). Kosushkin suggests that these TSD regions may be responsible for the tandem repeats in the A-tails [33]. Several studies have confirmed that the (AAAT)_n_ repeat polymorphism in the Alu sequence of the Neurofibromatosis-1 (*NF1*) gene is related to racial differentiation in autism patients and has a regulatory effect on gene expression [34,35,36]. These findings suggest that the AAAT repeat sequence carried by the SINE transposon may affect gene expression.

## 5. Conclusions

Based on bioinformatics and experiments, the SINE insertion of the first intron of the *CA5B* gene in the pig genome showed a relatively rich polymorphism in the nine pig breeds that were tested. We found that the SINE insertion may affect the thickness of back fat in Large White pigs and promote the expression of adipose tissue (back fat and leaf fat) in pigs. Additionally, our data suggested that the SINE insertion may enhance the core promoter activity of the *CA5B* gene. Furthermore, in the genomes of pigs of different breeds or individuals, the SINE insertion of the RIP-*CA5B* locus carries different numbers of (ATTT) repeats, which results in different lengths of SV.

## Figures and Tables

**Table 1:** Sample information for PCR verification of the SINE RIP.

Type	Breed	Province	Sample Size	Breeds Used in Creation of a Given Breed
Lean and hybrid	Sushan	Jiangsu	32	Meishan, Erhualian, and large white
Lean	Duroc	Anhui	24	/
Lean	Large White	Anhui	506	/
Lean	Landrace	Anhui	24	/
Medium and hybrid	Sujiang	Jiangsu	24	Jiangquhai, Fengjing, and Duroc
Fat	Meishan	Jiangsu	24	/
Fat	Fengjing	Jiangsu	24	/
Fat	Erhualian	Jiangsu	24	/
Miniature	Bama	Guangxi	24	/
Miniature	Banna	Yunnan	24	/
Miniature	Wuzhishan	Hainan	24	/
/	Wild boars	Anhui	24	/

**Table 2:** Polymorphism detection results of the SINE insertions in different breeds.

Breed	Sample Size	Genotype Number			Genotype Frequency			Allele Frequency		Hardy–Weinberg Equilibrium Test/*p* Value	PIC
		+/+	+/−	−/−	+/+	+/−	−/−	+	−		
Duroc	24	10	6	8	0.42	0.25	0.33	0.54	0.46	0.015	0.373
Landrace	24	13	11	0	0.54	0.46	0.00	0.77	0.23	0.145	0.291
Large White	24	18	3	3	0.75	0.13	0.13	0.81	0.19	0.004	0.258
Sujiang	24	17	5	2	0.71	0.21	0.08	0.81	0.19	0.121	0.258
Fengjing	24	15	8	1	0.63	0.33	0.04	0.79	0.21	0.959	0.275
Sushan	32	5	8	19	0.16	0.25	0.59	0.28	0.72	0.031	0.323
Meishan	24	17	7	0	0.71	0.29	0.00	0.85	0.15	0.403	0.218
Erhualian	24	9	14	1	0.38	0.58	0.04	0.67	0.33	0.126	0.346
Bama	24	21	2	1	0.88	0.08	0.04	0.92	0.08	0.026	0.141

Note: PIC: Polymorphic information content.

**Table 3:** Correlation Analysis of RIP-*CA5B* Insertion and Growth Performance of Large White Pigs.

Animal Numbers	Genotype	Genotype Frequency	Allele Frequency		Age at 100 kg Body Weight (Day)	Correcting Back Fat Thickness (cm)
			+	−		
316	+/+	0.66	0.80	0.20	163.25 ± 0.52	11.11 ± 0.14 ^a^
137	+/−	0.28			162.10 ± 0.52	11.00 ± 0.22 ^ab^
29	−/−	0.06			160.79 ± 1.07	10.15 ± 0.31 ^b^

Note: There is no significant difference between the values marked with “a” compared to the marked with “b”. However, there is a significant difference between the values marked with “a” compared to the marked with “b”.

**Table 4:** Length variations of SINE insertion in different breeds and individuals.

Breed-Individual Number	SV Length	Breed-Individual Number	SV Length	Breed-Individual Number	SV Length	Breed-Individual Number	SV Length
Duroc-1	308 bp	Sujiang-1	330 bp	Bama-1	314 bp	Tibetan-1	314 bp
Duroc-2	308 bp	Sujiang-2	314 bp	Bama-2	314 bp	Tibetan-2	314 bp
Duroc-3	308 bp	Sujiang-3	308 bp	Bama-3	314 bp	Tibetan-3	314 bp
Duroc-4	308 bp	Sujiang-4	308 bp	Bama-4	314 bp	Tibetan-4	314 bp
Duroc-5	308 bp	Sujiang-5	308 bp	Bama-5	314 bp	Tibetan-5	314 bp
Duroc-6	308 bp	Sujiang-6	308 bp	Erhualian-1	330 bp	Landrace-1	308 bp
Large white-1	314 bp	Sujiang-7	308 bp	Erhualian-2	330 bp	Landrace-2	313 bp
Large white-2	308 bp	Sujiang-8	308 bp	Erhualian-3	330 bp	Landrace-3	313 bp
Large white-3	308 bp	Sujiang-9	308 bp	Erhualian-4	308 bp	Landrace-4	308 bp
Large white-4	308 bp	Sushan-1	330 bp	Erhualian-5	330 bp	Landrace-5	308 bp
Large white-5	308 bp	Sushan-2	330 bp	Erhualian-6	330 bp	Landrace-6	308 bp
Large white-6	308 bp	Sushan-3	330 bp	Meishan-1	330 bp		
Large white-7	308 bp	Sushan-4	308 bp	Meishan-2	330 bp		
Large white-8	308 bp	Sushan-5	308 bp	Meishan-3	330 bp		
Large white-9	308 bp	Sushan-6	308 bp	Meishan-4	330 bp		
Large white-10	308 bp	Sushan-7	308 bp	Meishan-5	330 bp		
Large white-11	308 bp	Sushan-8	308 bp				
		Sushan-9	308 bp				

**Table 5:** Summary of information on changes in SINE insertion length of different varieties and individuals.

SV Length	Number of Individuals									(ATTT)_n_ *	total	% *
	Duroc	Large white	Sujiang	Sushan	Bama	Erhualian	Meishan	Tibetan	Landrace			
330 bp	0	0	1	3	0	5	5	0	0	(ATTT)_9_	14	23%
314 bp	0	1	1	0	5	0	0	5	0	(ATTT)_6_	12	19%
313 bp	0	0	0	0	0	0	0	0	2	(ATTT)_6_	2	3%
308 bp	6	10	7	6	0	1	0	0	4	(ATTT)_4_	34	55%
total	6	11	9	9	5	6	5	5	6	/	62	100%

* (ATTT)_n_: Types of ATTT repeats sequence carried by SINE insertion at RIP-*CA5B* locus. * %: Percentage of individuals with SV of the same length in all sequenced pigs.

## Data Availability

All data needed to evaluate the conclusions in this paper are present either in the main text or the Supplementary Materials.

## Supplementary Materials

The following supporting information can be downloaded at: https://www.mdpi.com/article/10.3390/ani13121942/s1, Figure S1: genotype distribution in different breed; Table S1: Primer information.
