# Supplementary material for: SINE Insertion in the Pig Carbonic Anhydrase 5B (CA5B) Gene Is Associated with Changes in Gene Expression and Phenotypic Variation
Source: Animals (Basel). 2023 Jun 9;13(12):1942. doi: 10.3390/ani13121942 (PMC10295633; doi:10.3390/ani13121942)
Supplement: Supplementary file 1 [file animals-13-01942-s001.zip › animals-2323169-supplementary.pdf]

Table S1. Primer information

Items	Forward primer	Reverse primer
Pool DNA genotype test	ACCTGAGTCACGTGTAGACAA	CCTTGAATGTGCCAGACGAAC
RIP verification and genotype in different breeds	CTTGACTTGGCATCAACTTCC	AAGGCACTGTTCTAGGCTCA
RT-qPCR test	ACAAAACCCGGAACCGAGA	AGTACCCGTTATTCCAGACGTG
$CA5B^{\text{SINE+783bp}}$/$CA5B^{\text{SINE-453bp}}$	ggtaccCTTGACTTGGCATCAACTTCC	acgcgtTAAGGCACTGTTCTAGGCTCA
$CA5B^{\text{SINE-453bp}}$	ggtaccCTCTCCTGAGCAATTTATTTATTT	gctagcGAATTTGGAGTGCCAAAATG
$CA5B^{\text{SINE-59bp}}$	ggtaccAAAAATGCAACACCCCCTTC	gctagcGAATTTGGAGTGCCAAAATG
$CA5B^{\text{SINE+}}$	ggtaccATTTATTTATTTATTTATTTATTT	acgcgtTAAATAAATTGGGAGTTCCTGTT
CA5B-pro1	acgcgtATTCTCCTTTCAATAAATTCCCCTTTTCC	aagcttTATGAAGTTCACCGTGACCCTAAGA
CA5B-pro2	acgcgtAACTAATTTTTTAAACGAGGCTCTTAAGC	aagcttACAGCTGTCCCGGCC
$(ATTT)_n$ sequencing primer	CTTGACTTGGCATCAACTTCC	AAGGCACTGTTCTAGGCTCA

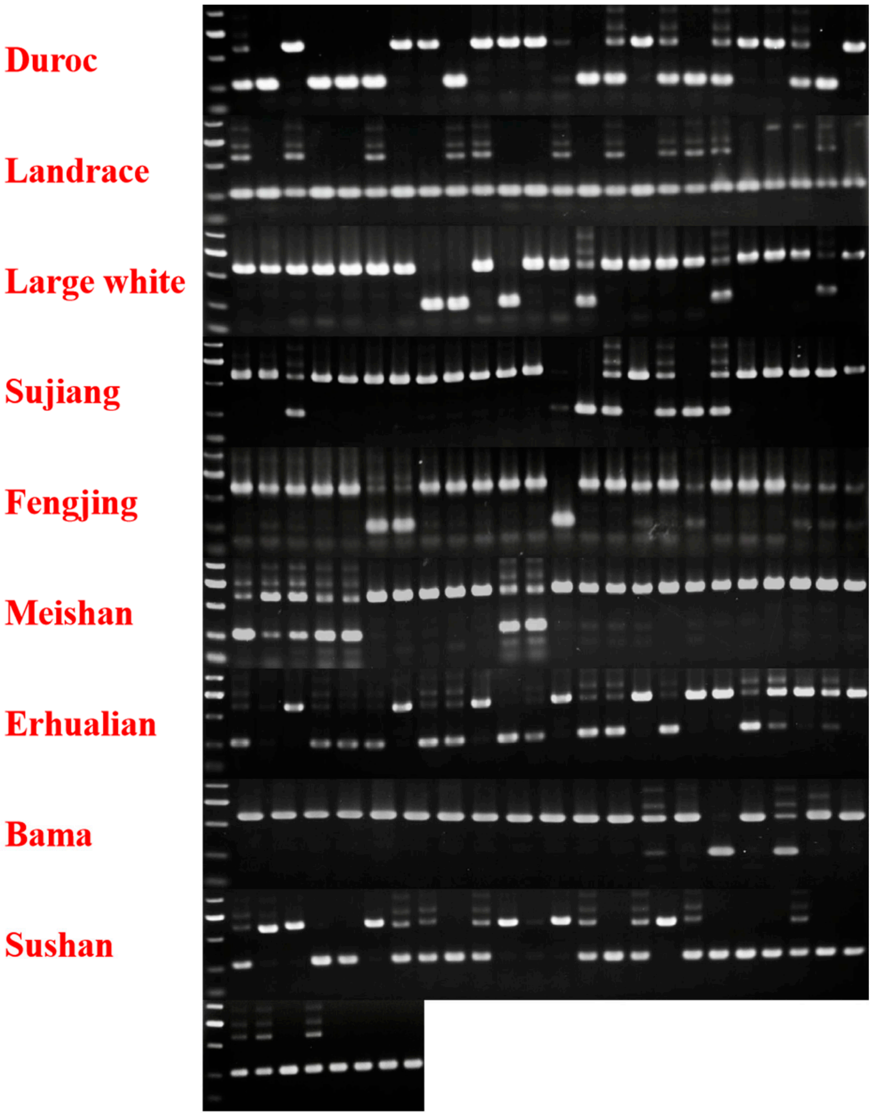

Figure.S1 genotype distribution in different breeds
